# How Open Data Shapes In Silico Transporter Modeling

**DOI:** 10.3390/molecules22030422

**Published:** 2017-03-07

**Authors:** Floriane Montanari, Barbara Zdrazil

**Affiliations:** Pharmacoinformatics Research Group, Department of Pharmaceutical Chemistry, University of Vienna, A-1090 Vienna, Austria; floriane.montanari@univie.ac.at

**Keywords:** transport proteins, computational modeling, open data, data curation, machine learning, multi-label classification, applicability domain

## Abstract

Chemical compound bioactivity and related data are nowadays easily available from open data sources and the open medicinal chemistry literature for many transmembrane proteins. Computational ligand-based modeling of transporters has therefore experienced a shift from local (quantitative) models to more global, qualitative, predictive models. As the size and heterogeneity of the data set rises, careful data curation becomes even more important. This includes, for example, not only a tailored cutoff setting for the generation of binary classes, but also the proper assessment of the applicability domain. Powerful machine learning algorithms (such as multi-label classification) now allow the simultaneous prediction of multiple related targets. However, the more complex, the less interpretable these models will get. We emphasize that transmembrane transporters are very peculiar, some of which act as off-targets rather than as real drug targets. Thus, careful selection of the right modeling technique is important, as well as cautious interpretation of results. We hope that, as more and more data will become available, we will be able to ameliorate and specify our models, coming closer towards function elucidation and the development of safer medicine.

## 1. Introduction: Computational Modeling as a Prosperous Strategy to Predict Ligand–Transporter Interactions

Transmembrane transporters are known to interact with many small molecules, conferring an altered pharmacokinetic behavior, drug resistance, and drug–drug interactions [1]. Thus, in silico modeling for the prediction of transporter interaction profiles is an effective strategy to identify potential adverse effects caused by such transport proteins at an early stage in the drug discovery and development pipeline.

Retrospective analyses of the large body of small compound bioactivity data in the biomedical literature and in open data sources help to increase the knowledge on certain peculiarities of the respective transporters. Nowadays, in a data-driven research environment, two sorts of ligand-based modeling approaches have won recognition: (1) the first approach tries to maximize the predictive chemical space of the model, going towards a “universal model” for a certain transporter; (2) the second approach tries to understand the driving features for a reduced ligand set to bind to the transporter of interest, or even tries to identify drivers for selectivity.

Irrespective of the different aims a predictive transporter model is built for, the quantity as well as the quality of the available data will tremendously influence model outcome and all conclusions drawn from there. Therefore, the open data revolution has brought tremendous opportunities to the drug discovery community on one hand, as well as new hurdles on the other hand. This review shall provide a critical overview of these hurdles, and it shall give guidelines for managing expectations depending on the underlying resources and methods used. Besides, it also can serve as a source of inspiration, reflecting on the promising methods we are having at hand nowadays.

## 2. Data, Data Everywhere

The availability of data for building ligand-based in silico transporter models is no longer a major limitation for many transport proteins. Open data sources for small compound transporter bioactivity data include specialized transporter databases like TP search [2], Metrabase (Metabolism and transport database) [3] and UCSF-FDA TransPortal [4], as well as broad compound collections like ChEMBL [5] (e.g., 9.8% of compounds in ChEMBL_22 report measurements on a transporter or ion channel), Open PHACTS [6], or PubChem [7].

In addition to compound bioactivity measurements, other layers of data shall inform transporter modeling, such as the Transporter Classification Database (TCDB) [8] (which provides over 10,000 human protein sequences along with their functional and phylogenetic classification), TransportDB 2.0 [9] (a bioinformatic pipeline to identify and annotate complete sets of transporters in any sequenced genome), and the University of California San Francisco (UCSF) pharmacogenetics database (which provides information on genetic variants in membrane transporter genes).

However, when it comes to the integration of diverging levels of data (gene expression data; pathway data; disease, functional, or phenotypic annotations, etc.), in order to tackle the biological research question, the close collaboration with experts from the relevant fields becomes inevitable, because, typically, researchers are only experienced in handling and interpreting one/a few type(s) of data.

Still, even staying in the compound-target-bioactivity space, we have to deal with different levels of data granularity in the open domain. In ChEMBL, for instance, curation of medicinal chemistry literature led to a well-structured collection of bioactivity data with assay descriptions included. These assay descriptions, however, follow no systematic classification other than determination of the assay type, assay format, and cell-line/tissues used [10]. Thus, it is not possible to map identical assays on the basis of this narrative description, unless information on the assays’ setup in the original primary literature is studied closer. This is especially crucial for transporters, where many different assay types are used both for evaluating transport and inhibition. For human P-glycoprotein, we have taken the effort to manually map those assays available in a former version of ChEMBL [11]; however, as the body of data increases, newer data needs to be mapped accordingly. In our opinion, a specialized transporter assay ontology would aid towards the easier curation and mapping of transporter bioactivity data [12]. A more recently launched transporter data repository, Metrabase, contains “action type” annotations (substrate/non-substrate, inhibitor/non-inhibitor, repressor, stimulator, inducer/non-inducer) which were retrieved from literature or other open data sources (e.g., TP-search, ChEMBL). However, these annotations have to be used with caution, as some might depend on certain cutoffs used for their determination (e.g., inhibitor/non-inhibitor). Although all data points are linked to either a literature reference or a database, a major weakness of this data source is that assay descriptions, cell lines, compound concentrations, substrates used, etc. are missing. Therefore, the user would need to rely on the processed data provided by the database curators in order to use it for modeling purposes. In addition, this sort of binary data (e.g., substrate/non-substrate; inhibitor/non-inhibitor) can consequently only be used for binary classification models, as discrete bioactivity values are not indicated. As the more detailed level of assay descriptions is not given, their mapping is totally out of reach when using this source.

It appears essential, given the diverse sources of data and assay annotation granularity, to establish an adequate data curation process for transporter pharmacological data.

## 3. What Is Proper Data Curation?

In the field of cheminformatics, we are largely dealing with compound bioactivity data. Since open data sources in that domain mostly report data extracted from the medicinal chemistry literature, large compound collections measured under the same assay conditions are rather rarely available. This is especially true for transporter substrates. Transport inhibition data is more likely screened in one sample, since in such a case a whole panel of compounds can be screened at once for their potential to inhibit the transport of a known substrate [13].

In order to build up a predictive model for a certain transporter, the data always must be sent through a rigorous filtering and pre-processing pipeline for creating consistent, high-quality data sets. Notably, there is no consistent opinion within in the cheminformatics and drug discovery community on the hallmarks of proper data curation.

Regarding chemical structures, a set of rules was detailed in a paper by Fourches et al. [14], including removing inorganic compounds, stripping salts, normalizing chemotypes, and so on, with a recent update/extension for chemogenomics data, where the same authors propose a chemical and biological data curation workflow [15].

The need for (semi-)automated workflows for data extraction and curation has obviously been identified by a plentitude of research groups in the field of computational modeling. In 2014, for instance, Anger et al. proposed a standardized routine for heterogeneous input data complemented by a modeling workflow for off-target prediction [16]. Similarly, we merged inhibitor data from different literature sources for the human serotonin and dopamine transporter (hSERT; hDAT) by also using a workflow routine (KNIME [17]). However, the aim of our study was the retrieval of overlap compounds and study of selectivity trends [18]. We found that setting cutoffs for labeling actives/inactives is a crucial, non-trivial task which is better tailored to the specific transporter and bioactivity endpoint under study. Indeed, the right cutoff might depend on the predicted endpoint, as also identified by other researchers in the field: for the prediction of toxicity or in vivo endpoints of transporters, for instance, much higher thresholds were proposed than for labeling activity/inactivity with respect to affinity. For example, for bile salt export pump (BSEP; ABCB11), an overall in vitro IC_50_ “threshold of toxicological concern” of 300 µM was proposed [19].

## 4. Transporter Modeling: Earlier and Now

In previous decades, a lot of effort was put towards the design and synthesis of ABC-transporter inhibitors due to the involvement of some of these proteins in cancer multidrug resistance [20]: P-glycoprotein (P-gp; MDR1/ABCB1), breast cancer resistance protein (BCRP; ABCG2), and multidrug resistance proteins (MRP1–4) were such transporters of interest. As a result, earlier computational models focused on quite narrow chemical compound classes. Examples are manifold: Ecker et al. performed a quantitative structure–activity relationship (QSAR) study on benzofuran analogs for P-gp inhibition [21]; Li et al. did 3D-QSAR on steroids (which can either be substrates or inhibitors of P-gp) [22]; Zhang and colleagues built QSAR models of 25 flavonoids to predict their BCRP inhibition [23]; and Müller et al. proposed a 3D-QSAR model for predicting the pIC_50_ against P-gp of a series of tariquidar analogs [24]. These models usually consist of extracting molecular descriptors from the compounds of interest (2D descriptors for regular QSAR or 3D descriptors for 3D-QSAR) and then linearly combining them to predict a continuous endpoint like pIC_50_. Models able to predict a continuous value are referred to as regression models.

In these earlier days of in silico transporter modeling, chemical compound series tended not only to be congeneric (core molecule with varying substituents), but also concise and measured in one assay sample (or at least by using the same assay protocol). Therefore, building quantitative models predicting the half maximal inhibitory/effective concentration (IC_50_ or EC_50_) of structurally related compounds is eligible here. Such regression models proved useful for gathering a deep understanding about a certain chemical compound class with respect to its interaction with a certain transporter, and they are suited to inform future synthetic efforts [25]. However, these models are not suited for predictions beyond the narrow chemical space of the training set. Therefore, even in these early days, certain efforts to build consensus models were undertaken by integrating local models from multiple chemical series [26].

**Figure 1:**
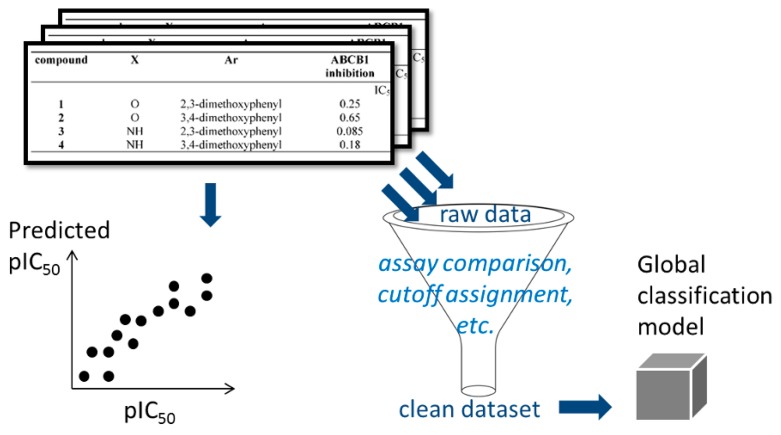
Schematic depiction of data compilation and modeling workflow.

More recently, the transporter field has seen a rise in publications of qualitative models (also referred to as classification models, they are able to predict classes of activity, such as “inhibition”) built on a diverse set of chemical structures, collected from open data sources (Figure 1). In 2011, Broccatelli and colleagues merged P-gp inhibition data from 44 publications reporting both IC_50_ and percentage of inhibition, applying clear cutoffs and removing compounds with medium activity or disagreeing labels [27]. The final training set used contained 772 compounds and their final model, and combining 3D-QSAR and linear discriminant analysis led to what is today one of the most predictive models for P-gp inhibition (accuracy of 0.86 in external validation). Similarly, we collected data for BCRP inhibition from 47 sources (publications and PubChem bioassays) and applied assay-tailored thresholds to distinguish inhibitors from non-inhibitors [28]. This led to a data set encompassing a broad chemical space, containing 978 annotated compounds. The best model generated on the basis of this data set was then used as a virtual screening tool to identify new inhibitors of BCRP [29]. Going a step further, Kotsampasakou and colleagues built up consensus classification models for OATP1B1 and OATP1B3 inhibition based on 1700 compounds from the open medicinal chemistry literature [30]. An ensemble of random forest and support vector machine models was built upon different descriptor sets, and a consensus score finally determined whether a compound is likely an inhibitor of these transporters or not.

Summarizing the changes that the open data revolution has brought with respect to increasing sizes of training sets for in silico modeling, it seems that nowadays more powerful machine learning methods can be used, leading to models that are more predictive and more likely to generalize, going towards global predictive models. Of course, this comes with the side effect of losing interpretability and clear guidelines for medicinal chemists. Another risk is increasing the noise in the models by including too diverse data (in terms of assay variability, cutoff uncertainty, etc.). However, such global models have proven useful in virtual screening applications [29,30,31] and can definitely be used to quickly flag potentially problematic compounds in very early phases of drug discovery.

A parallel trend to the one just described (going from local to more globally predictive models), was influenced by the fields of chemical biology and systems biology. There, the tendency in model generation clearly points towards the generation of holistic models, such as models for polypharmacology prediction and network-based approaches [32,33,34,35]. In the field of transporter modeling, researchers have recently tried to approach the problem of predicting the interaction of compounds with a whole range of locally coexpressed transporters (at an organ or barrier level). That way, better knowledge of the fate of a compound when entering such an organ can be gathered. For example, Sedykh and coworkers built an ensemble of QSAR models to predict the inhibition of, and transport by a range of transporters expressed in the gastrointestinal tract (P-gp, BCRP, MRP1–4, apical sodium-dependent bile acid transporter (ASBT), organic anion transporter polypeptide (OATP)2B1, organic cation transporter 1 (OCT1), and monocarboxylate transporter 1 (MCT1)) [36]. The authors collected substrate and inhibition data from many public sources and collated their results using a “confidence score”: a compound with many agreeing pharmacological annotations will have a very high confidence score. The individual data sets contained between 0 (MCT1 substrates) and 1571 compounds (P-gp inhibitors), and predictive models were built only for data sets of a certain size. Twenty consensus models in total were built with cross-validation accuracies ranging between 72% and 100%.

Developing the idea of combining several transporter targets further, researchers have tried to use information on one transporter to help predict activity on related transporters using multi-label classification. Recently, we proposed an automatic workflow to collect multi-label data from the Open PHACTS discovery platform and combine it with in-house or manually curated data [37]. Using inhibition data of P-gp and BCRP, we tried to simultaneously model inhibition on both transporters using different methods. One of them (a multi-class classification tree) allowed us to gain insights into the driving features for selectivity of inhibitors for one transporter over the other. Aniceto and colleagues used the same methods to predict P-gp, BCRP, MRP1, and MRP2 substrates with data from Metrabase [38]. In both cases, little improvement in model performance was found when taking into account the overlap between pharmacological data (using classifier chains [39]), however we still believe that this method might prove useful in the future, especially when it comes to simultaneous modeling of many targets (i.e., adding more labels to the problem). Table 1 summarizes the methods used in the previously cited works.

While QSAR modeling on congeneric series benefits from the simplicity of their training data (both in terms of bioassays and chemical space), new approaches, facilitated by open bioactivity databases, have risen. There, by aggregating data, researchers may obtain broader coverage of chemical space and use powerful machine learning methods. Of course, bringing together data from different sources comes with its own hurdles, some of which are addressed in the following section.

## 5. Chemical Space, Analog Bias, and Applicability Domain

In all our investigations, the chemical model space must be analyzed thoroughly. In earlier days, at the beginnings of Hansch analysis [40] and QSAR, chemical compound series tended to be concise and congeneric, hence the applicability domain (AD) of these models was obvious.

Nowadays, by combining data from different medicinal chemistry sources, the training sets of global transporter models cover a broader space but in a very irregular way. Indeed, given scaffolds will be overrepresented, a characteristic of data sets referred to as “analog bias”. This complicates both the assertion of an applicability domain and the unbiased validation of the models, since random splitting and cross-validation (both common splitting methods to assess the reliability of a model) might cause overoptimistic results [41]. Since the scarcity of data for some transporters does not allow for building large, independent test sets, we suggest the iterative use of sets of entire sources as external test sets instead of random sets, in a leave-sources-out cross-validation fashion (unpublished work). Broccatelli and colleagues indeed used an external set made up of a separate set of sources to evaluate their P-gp inhibition model [27]. Such a splitting method may allow a more stringent and accurate evaluation of the model performance on new data, and can resemble a time-split as routinely used in the industry.

Regarding applicability domain-filtering methods, a plethora has been proposed, yet no single method seems to be useful in all cases. Carrió and coworkers recently proposed a consensus score for applicability domain analysis (ADAN) [42]. This method combines distances to the training set, outlier score, similarity of the prediction to similar compounds, and prediction error to similar compounds to assess the reliability of a prediction. Unfortunately, this method was developed for regression models and needs to be adapted for classification models. While a detailed review of applicability domain methods is not in the scope of this work, a new approach by Aniceto and colleagues is worth mentioning [43]. Their method, named reliability-density neighborhood (RDN), combines the density and the reliability of nearby training instances into one metric. This method has not been widely applied yet, but may be a good way to handle unequally covered chemical space in the training set of classification models.

While the problems of analog bias and applicability domain are recurrent in computational chemistry, they are especially important to tackle for transporter models. Indeed, transporters are mostly rather off-targets than regular drug targets (like kinases or G-protein-coupled receptors (GPCRs)), and therefore little high-throughput data is available for most transporters. As a result, the training sets are gathered from individual publications of small molecule families and the resulting chemical space becomes especially irregular.

## 6. The Future: New Areas to Explore

Recent advances in protein structure elucidation, like cryo-electron microscopy, will increase our knowledge of membrane transporters. In 2012, Ekins and colleagues proposed combining automatically generated homology models of the different transporters as well as classical QSAR studies to improve both model predictivity and biological understanding [44]. Pioneer work of structure-based classification using the docking score has already been published for P-gp [45], without, however, surpassing the performance of ligand-based models. One could think that including docking scores, pharmacophore fits, and/or interaction fingerprints into the feature matrix used in ligand-based modeling would bring an edge over simple molecular descriptors.

These kind of approaches have already been used successfully for kinases [46] and GPCRs [47,48], and could be adapted to the field of transporter interactions as soon as reliable structural models become available.

Beyond looking at transporters one by one, and constantly trying to improve individual models, it may be worth examining them as an ensemble. Indeed, transporters frequently co-express at organs and barriers that are crucial for drug distribution, metabolism, and disposition like the blood–brain barrier, the liver, the kidneys, among others. Individual impacts of the inhibition of some transporters by drugs have been evaluated and can be linked to toxicity. For example, inhibition of the bile salt export pump (BSEP/ABCB11) at the canalicular membrane of hepatocytes may lead to cholestasis [49]; inhibition of OATP1B1 and OATP1B3 at the basolateral membrane of hepatocytes may lead to hyperbilirubinemia [50]. However, the in vivo toxicological effects are very complex and may involve many transporters and enzymes, with potential compensatory effect. One way of trying to predict toxicological endpoints is using binary predictions on a range of transporters as fingerprints combined with regular molecular descriptors. Such an approach was recently published for hyperbilirubinemia [51]. While the authors saw no improvement when adding OATP1B1 and OATP1B3 predictions to their feature matrix, it is possible that adding more hepatic transporters would have a positive impact on the final model. Many other liver toxicity endpoints could be approached using that kind of methodology, providing that there are solid predictive models for all the transporters involved in the studied process.

Another promising integrative approach was recently developed within the pharmaceutical industry. It describes the use of biological fingerprints/signatures for modeling and screening purposes [52,53]. These fingerprints can be used as input vectors for modeling reflecting target-based and phenotypic biological effects. Although there were efforts to develop these within the open domain using only PubChem’s bioassay data [52], high-throughput screening (HTS) data on transporters, as already mentioned, is rare. It is tempting to speculate that these kind of data will be in high demand in the future, as also shown by some technological advancements in this field [54,55].

One problem that is frequently faced by researchers studying transporters is the lack of pharmacological data for some particular transporters. For example, MDR3 (gene ABCB4) has only six bioactivities reported in ChEMBL_22. This protein, however, plays a role in bile salt homeostasis by flopping phosphatidylcholine molecules to the outer membrane leaflet in hepatocytes, where they are picked up to form mixed micelles with the bile salts. Malfunction of MDR3 may lead to bile duct toxicity or cholangitis [56]. The lack of pharmacological data for this protein precludes its use in a global model of drug-induced liver injury. To tackle this problem, computational chemogenomics can be applied [57]. Concretely, a frequently used computational chemogenomic approach includes physicochemical descriptors for the amino-acid sequences of a whole family of proteins along with bioactivity data. A model can then be built to predict the activity of a compound for a specific target given its chemical descriptors and the target sequence descriptors. Such a method has been used to de-orphanize GPCRs [58], but also for enzymes and ion channels [59]. The main advantage of this method is that it does not require a 3D structure of the target of interest nor existing ligands. However, the group of A. Bender also showed a benefit in model performance for such proteochemometric approaches for cases where large amounts of chemical data was available for all targets [60].

Support vector machines and other kernel methods have been frequently used to learn these chemogenomics tasks, but, interestingly, Erhan and colleagues [61] presented a neural network with two hidden layers, in which the first hidden layer learns the biological representation (using, for example, binding site pocket fingerprints as input), while the second layer handles both the biological information and the chemical representation. The model was built on large HTS results for 24 targets, each target having between 1000 and 14,000 compounds with known activity. Overall, their results were not as good as those obtained with a kernel method, but still the approach is worth further investigations.

Perhaps going for a deeper architecture better capable of learning both the biological and chemical spaces would prove useful. Adding more layers and parameters of course requires a lot of control over overfitting and a lot of training data, so this can presently be seen only in collaboration with the pharmaceutical industry. Competitions like the Merck Kaggle challenge in 2012 released large data sets for many different targets (cytochromes, GPCRs, hERG channel, etc.), and the winner was a deep neural network tackling all the tasks at once [62]. Here again, the individual 30 tasks had between 2092 and 318,000 compounds, the smaller tasks benefitting from the parameters learnt in the larger tasks. In our opinion, such powerful methods could be applied to the transporter field as well, as soon as enough data is released.

## 7. Conclusions

Transporters are very peculiar targets. Indeed, they are often extremely promiscuous and cannot be defined by a single binding site. In addition, different transporters co-expressed at the same organs have overlapping substrate and inhibitor profiles and seem to work in a semi-redundant fashion [1]. Trying to predict the effect of small molecules on these transporters is both a worthwhile and needed effort, in order to further elucidate their biological function. Indeed, even if some of them are not considered as drug targets anymore, they have an increasingly recognized impact on the pharmacokinetics of xenobiotics and must be taken into account during drug development.

In this review, we highlighted useful open data sources for transporters, gave recent examples of timely transporter modeling, and critically discussed existing hurdles during this process. While more and more data becomes available, it is critical to select the right methodology for the biological question at hand, since all methods have their strengths and limitations. While it can be useful to have a good universally applicable predictive model in some cases, it might be more useful to be able to interpret results and learn from the distinguishing features another time. In all cases, proper data curation is an absolute must. However, no consistent rules among the community can guide these critical pre-processing steps. Well-known problems in data curation are even more pronounced in the transporter field: bioassay samples in the open domain are rather small and manifold; contradictive or unclear data annotation is part of the game (e.g., inhibitor, substrate, inducer etc.); and the publicly available data is plagued by analog bias.

While it is promising that some consensus modeling techniques (e.g., for toxicity prediction of in vivo endpoints) and multi-label classification techniques are nowadays also applied to the transporter field, we are still far away from the full exploitation of more complex methods (such as deep neural networks), which certainly demand for a lot more data points than we have currently available for transport proteins.

Unraveling some of the secrets of transporter mediated drug–drug interactions and drug resistance by making use of these powerful methods would definitely help bring us a step closer towards safer medicines.

## Figures and Tables

**Table 1:** Summary of the methods used to predict ligand–transporter interactions.

Reference	Endpoint	Training Set	Method Type	Algorithm
Ecker [21]	P-gp inhibition	20 benzofurylethanolamine analogs of propafenone	regression	multiple linear regression
Li [22]	P-gp transport and inhibition	20 steroids	regression	3D-QSAR ^a^
Zhang [23]	BCRP inhibition	25 flavonoids	regression	feature selection and multiple linear regression
Müller [24]	P-gp inhibition	28 tariquidar analogs	regression	3D-QSAR ^a^
Pajeva [26]	P-gp inhibition	40 phenothiazines, thioxanthenes, and structurally related drugs	regression	3D-QSAR ^a^
Broccatelli [27]	P-gp inhibition	772 diverse compounds	classification	PLS-DA ^b^ and LDA ^c^
Montanari [28,29]	BCRP inhibition	978 diverse compounds	classification	logistic regression
Kotsampasakou [30]	OATP1B1 and OATP1B3 inhibition	1700 diverse compounds	classification	ensemble of SVMs ^d^ and random forests
Sedykh [36]	Inhibition and transport for a range of intestinal transporters	up to 1571 diverse compounds	classification	random forest, k-nearest neighbors or SVM ^d^
Montanari [37]	P-gp and BCRP inhibition	2191 diverse compounds	multi-label classification	classifiers chain
Aniceto [38]	P-gp, BCRP, MRP1, and MRP2 transport	1493 diverse compounds	multi-label classification	classifiers chain

^a^ three-dimensional quantitative structure-activity relationship; ^b^ partial least-squares discriminant analysis; ^c^ linear discriminant analysis; ^d^ support vector machines.
